# The impact of parenting styles on depression among high-risk adolescents of different genders in chinese urban samples: the mediating effects of adverse childhood experiences and learning burnout

**DOI:** 10.3389/fpsyt.2025.1594584

**Published:** 2025-10-16

**Authors:** Peiqi Tang, Yuxuan Guo, Xiaoqiang Xiao, Juexi Li, Ting Pu, Ting Yang, Haixi Zuo, Xiaoxia Fan, Liyuan Li, Bo Zhou

**Affiliations:** ^1^ Sichuan Provincial Center for Mental Health, Sichuan Provincial People’s Hospital, University of Electronic Science and Technology of China, Chengdu, China; ^2^ Clinical Medical school, Southwest Medical University, Luzhou, China; ^3^ Clinical Medical school, North Sichuan Medical College, Nanchong, China

**Keywords:** depression, adverse childhood experiences, learning burnout, mediation, adolecence, adolescent

## Abstract

**Background:**

Parenting styles, adverse childhood experiences, and learning burnout are significant risk factors for depression in adolescents; however, the underlying mechanisms and gender differences remain inadequately explored. This study aims to investigate the mediating roles of adverse childhood experiences and learning burnout in the relationship between parenting styles and adolescent depression, as well as to examine potential gender differences.

**Methods:**

A total of 3,180 high-risk adolescents participated in this study. Key variables were assessed using standardized instruments, including the Short Egna Minnen av Barndom Uppfostran (s-EMBU), the Childhood Trauma Questionnaire-Short Form (CTQ-SF), the Learning Burnout Scale, and the Patient Health Questionnaire-9 (PHQ-9). Structural equation modeling analysis was performed using AMOS 24.0.

**Results:**

Adverse childhood experiences (*β* = 0.305, 95% CI [0.203, 0.420]) and learning burnout (*β* = 0.118, 95% CI [0.064, 0.183]) emerged as mediators between various parenting styles and depression. Among female adolescents, parenting styles influenced the onset of depression through both direct and sequential mediation involving adverse childhood experiences and learning burnout. Conversely, among male adolescents, overprotective parenting styles impacted depression through the mediating effect of learning burnout.

**Conclusion:**

Adverse childhood experiences and learning burnout mediate the relationship between different parenting styles and depression. Additionally, the pathways through which parenting styles affect depression demonstrate gender differences.

## Introduction

Adolescence, as a distinct period of physiological and psychological development, is characterized by significant cognitive and emotional changes. The interplay of numerous factors, including genetic predispositions and environmental influences, contributes to the complex etiology of emotional issues among adolescents (1). It is estimated that 14% of adolescents aged 10 to 19 experience mental health problems, with depressive symptoms being particularly prominent (2). Notably, the prevalence of severe depression among females is twice that of males (3, 4). This disparity is believed to emerge between the ages of 13 and 15 and to widen between 15 and 18, persisting into adulthood (5–7). The factors contributing to adolescent depression are multifaceted, encompassing childhood environments, adverse life events, personality traits, and social support (8–11). Among these, parenting styles within the developmental environment have been shown to exert a long-term influence on adolescent personality development and other psychological characteristics, making it one of the most significant factors affecting adolescent depression (12). Parenting styles consist of a range of stable attitudes expressed through specific goal-directed behaviors (such as educational practices) and non-goal-directed behaviors (such as emotional expression), which are closely related to an individual’s socialization process and psychological development (13). Perris et al. propose that parental attitudes and behaviors can be evaluated across three dimensions: rejection and denial, emotional warmth and understanding, and overprotection and interference (14). Negative parenting styles characterized by rejection and denial, as well as overprotection and interference, are associated with an increased risk of depression in adolescents (8, 15, 16). Conversely, parental emotional warmth and understanding are beneficial in reducing the risk of depressive symptoms among adolescents (17). Importantly, long-term negative parenting styles practices can lead to the development of different mental disorders in adolescents of varying genders. For instance, excessive maternal interference is linked to an increased risk of depression, panic disorder, post-traumatic stress disorder, and eating disorders, while excessive paternal interference is associated with a heightened risk of agoraphobia and alcohol abuse/dependence. Furthermore, among adolescents with high substance abuse, females report higher levels of maternal control, whereas males report higher levels of paternal control (18, 19). This may be attributed to the differing social expectations and role training that boys and girls receive, as well as the tendency of adolescents to imitate and identify with the behaviors and attitudes of their same-sex parents, leading to gender-specific differences in the prevalence of mental health issues (20). Additionally, the influence of parenting styles styles on adolescents varies across different cultural contexts. According to cultural dimension theory (18, 21), a male-centric culture exists in China, where boys are encouraged from a young age to be independent, confident, and assume the role of providers, thereby establishing deeper social connections. In contrast, girls are socialized to be obedient and sensitive, with a greater emphasis on interpersonal relationships and emotional experiences, often being expected to return to familial roles. Consequently, girls may be more sensitive to parent-child relationships. Thus, it is evident that the developmental challenges faced by adolescents of different genders are distinct, leading to varied outcomes influenced by negative parenting styles practices.

Although existing research has examined the relationship between parenting styles and adolescent depression, no studies have yet integrated a mediational model to elucidate how parenting styles impact the risk of depression in Chinese male and female adolescents through adverse childhood experiences and learning burnout.

### Adverse childhood experiences as a mediator

Adverse childhood experiences (ACEs) refer to the prolonged exposure of individuals during their formative years to detrimental environmental conditions characterized by instability and insecurity. Such adverse environments pose continuous threats, resulting in emotional deprivation and unmet needs. ACEs encompass five categories of childhood maltreatment: emotional abuse, physical abuse, sexual abuse, emotional neglect, and physical neglect, as well as five forms of familial dysfunction during childhood: parental separation/divorce, domestic violence, substance abuse, mental illness, and incarceration (22, 23). The emergence of ACEs is closely linked to the family environment, particularly parenting styles (24). When parents frequently employ negative parenting practices, such as denial and neglect, adolescents may develop a tendency to attribute negative events to themselves in a persistent manner, leading to self-defeating beliefs (e.g., “I always fail,” “I am not good enough”). Prolonged exposure to negative experiences can foster a sense of helplessness in adolescents, reinforcing maladaptive thought patterns and rendering them oblivious to positive or neutral information, thereby contributing to the onset of ACEs (25, 26). Notably, research indicates that the use of violence and harsh, indifferent educational practices during parenting can inflict significant emotional neglect, emotional abuse, and physical abuse on children (27). Conversely, children who experience adversity are more likely to report feelings of parental rejection, indifference, and overprotection (13, 28).

Studies have demonstrated that ACEs is associated with a range of chronic diseases, including depression, severe obesity, heart disease, cancer, liver disease, and chronic lung disease (29, 30). LeMoult et al. found that individuals with a history of ACEs are 2.5 times more likely to develop depression compared to those without such a history, with a greater number of ACEs correlating with more severe depressive symptoms (11). Furthermore, ACEs, particularly bullying and sexual abuse, may increase the risk of depressive symptoms in adolescents through heightened bodily inflammatory responses (31). In summary, ACEs may serve as a significant mediating factor in elucidating the impact of parenting styles on adolescent depressive symptoms.

### Learning burnout as a mediator

Learning burnout refers to a state of excessive depletion of physical and mental resources and energy exhaustion that arises from prolonged exposure to stress. It is positively correlated with psychological issues such as anxiety, depression, emotional instability, and obsessive-compulsive symptoms (32). In China, academic life constitutes a significant aspect of adolescents’ lives, compounded by substantial social competition pressures. Many parents impose high academic expectations on their children, often employing parenting strategies that restrict leisure activities and undermine perceived progress to motivate learning (33). As a result, some adolescents, enduring this high-pressure environment and experiencing low achievement satisfaction, gradually develop a sense of alienation from their studies and enter a state of learning burnout characterized by energy depletion. When adolescents encounter negative learning experiences, such as frustration, fatigue, dissatisfaction, and low self-esteem, these adverse psychological states can lead to detrimental changes in their emotions or cognition, thereby impacting their mental health (33). Consequently, learning burnout may serve as a mediating factor between parenting styles and depressive symptoms in adolescents.

### ACEs and learning burnout as a chain mediator

Based on an integrated theoretical framework of psychology and neuroendocrinology—chronic stress, dysregulation of the HPA axis, impaired brain plasticity, and a decline in academic functioning model—children who experience prolonged exposure to abuse and neglect exhibit persistent hyperactivity or hypoactivity of the hypothalamic-pituitary-adrenal (HPA) axis. This dysregulation leads to disruptions in cortisol rhythms, which in turn damage neural circuits in the hippocampus and prefrontal cortex responsible for memory, attention, and executive control, thereby hindering the acquisition of academic skills. Once academic difficulties manifest, negative feedback from failures in learning and adverse interactions with teachers can result in emotional exhaustion and avoidance of academic tasks, contributing to the emergence of learning burnout (22). A longitudinal study found that elevated cortisol levels in children aged 7 to 24 months could predict lower academic performance at age 5, while an increase of one point in the ACEs score during the first grade was associated with a 1.3 to 1.9-fold increase in the risk of subsequent academic failure (34). In summary, negative parenting styles and ACEs as early and chronic stressors in adolescence, potentially leading to learning burnout by affecting the HPA axis and brain structure.

**Figure 1:**
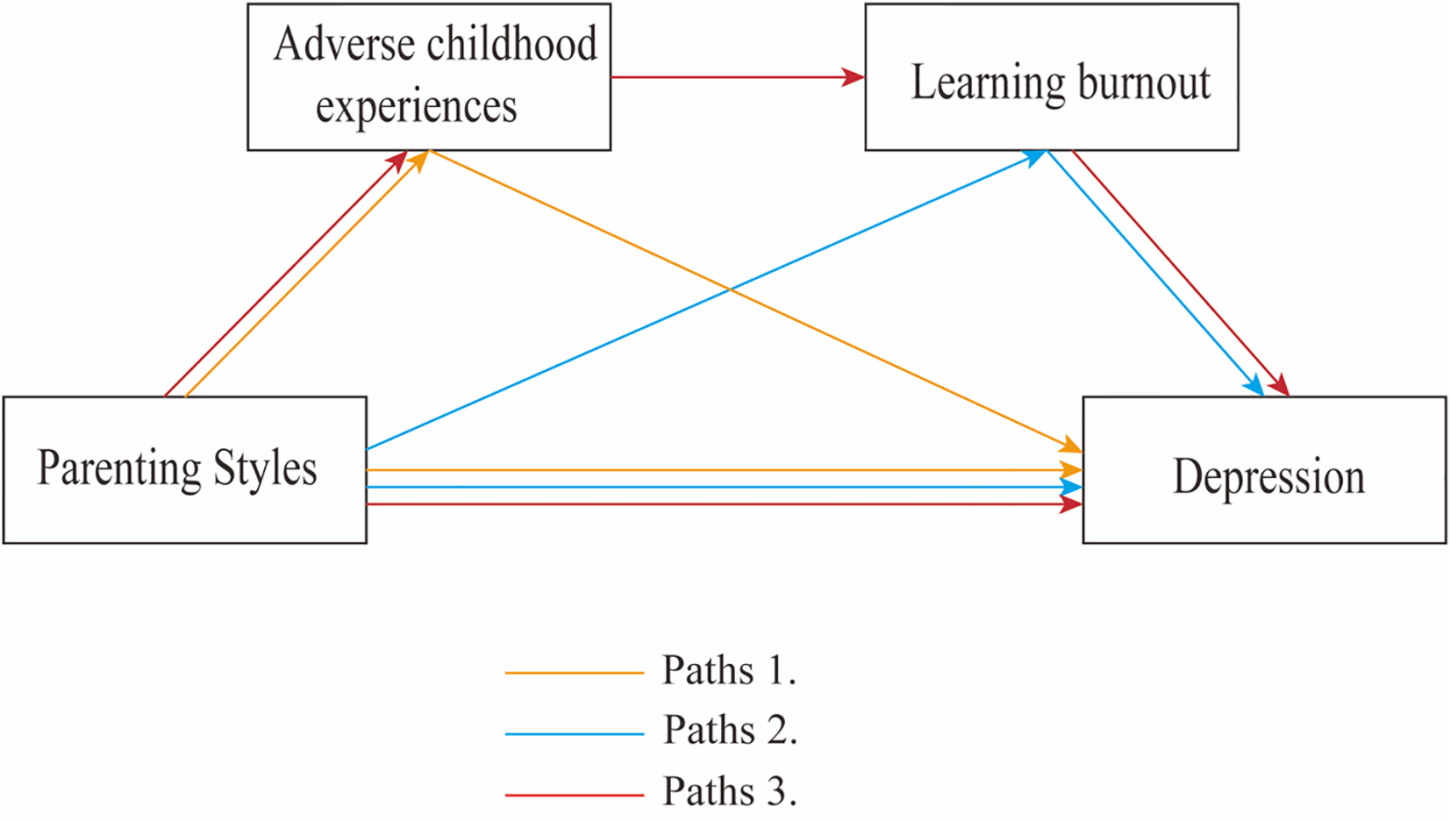
A hypothetical model of parting styles affecting depression.

This study aims to explore the chain mediating effects of ACEs and learning burnout between parenting styles and adolescent depressive symptoms, utilizing a large sample of adolescent data and employing structural equation modeling (SEM). The following hypotheses are proposed (see **Figure 1**): Hypothesis 1: ACEs and learning burnout mediate the relationship between parenting styles and depressive symptoms (Path 1, Path 2); Hypothesis 2: There exists a chain mediating effect of ACEs and learning burnout between parenting styles and depressive symptoms (Path 3); Hypothesis 3: There are differences in the parenting styles of fathers and mothers in relation to the pathways leading to adolescent depressive symptoms; Hypothesis 4: Gender differences exist in the pathways through which parenting styles influence adolescent depressive symptoms.

## Methods

### Participants and procedure

In response to the Ministry of Education’s initiative to enhance psychological assessments for students, the Education Bureau of a district in Chengdu, in collaboration with our institution, distributed psychological assessment questionnaires to all primary and secondary schools in the region in November 2021. The target population for the questionnaire included students from the fourth to the twelfth grade. Prior to the completion of the questionnaires, informed consent was obtained from both the students and their guardians. Additionally, our institution provided standardized training for 20 psychological educators from the collaborating schools, which included topics on privacy protection and emergency response. Those who passed the assessment subsequently served as primary examiners. The assessments were conducted by class, with students logging into a secure system using their identification numbers in a computer lab. The psychological educators read standardized instructions and emphasized the principles of anonymity and voluntary participation, while the homeroom teachers supervised the entire process. The system was equipped with mandatory validation features, allowing each IP address to submit responses only once. During the data screening phase, a dual quality control process was implemented: the system automatically filtered out invalid data, and manual reviews were conducted to exclude questionnaires with logical inconsistencies between reverse-scored and forward-scored items, as well as those exhibiting patterned responses. Through these measures, a total of 60,011 valid questionnaires were collected. This study received ethical review and approval from the Ethics Committee of Sichuan Provincial People’s Hospital and Sichuan Provincial Mental Health Center.

**Figure 2:**
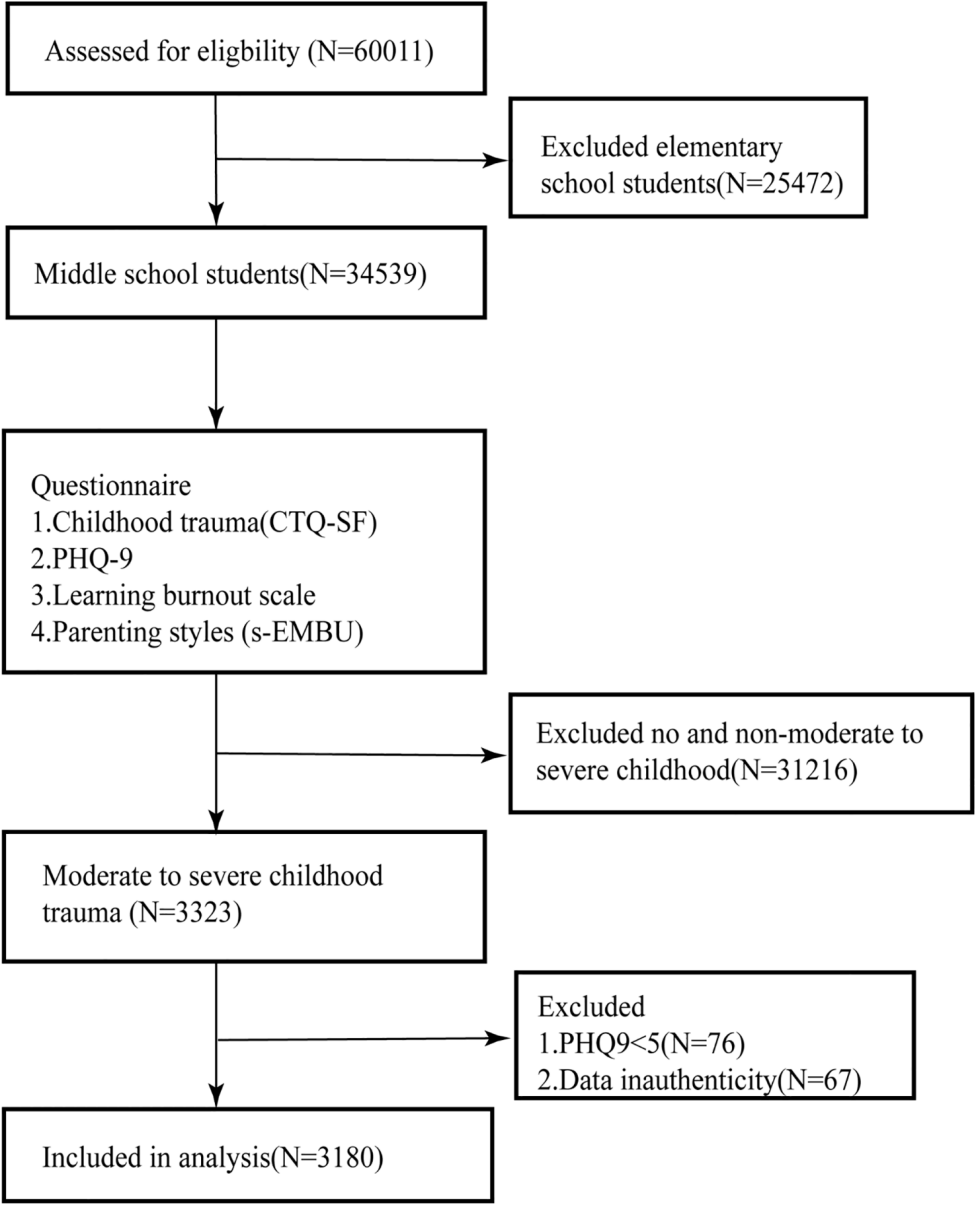
A flow diagram for screening research subjects.

From the 60,011 questionnaires, we identified 34,539 adolescents in the first year of junior high to the third year of senior high school. Subsequently, we utilized the Childhood Trauma Questionnaire-Short Form (CTQ-SF) to select 3,323 adolescents who experienced moderate to severe childhood trauma. Among these individuals, we further screened for those with a PHQ-9 score of ≥5, resulting in a final inclusion of 3,180 high-risk adolescents in this study (**Figure 2**).

### Measuring instrument

The Short Egna Minnen av Barndom Uppfostran (s-EMBU) questionnaire was employed to assess parenting styles (35). The s-EMBU consists of two versions: the father’s version and the mother’s version, each containing 21 items, resulting in a total of 42 items. It encompasses six dimensions: paternal rejection, maternal rejection, paternal emotional warmth, maternal emotional warmth, paternal overprotection, and maternal overprotection. Each item is rated on a 4-point scale ranging from 1 to 4, with items 29 and 30 scored in reverse. A higher score in a given dimension indicates a greater tendency of parents to exhibit that particular parenting style. This questionnaire has been widely utilized in research involving Chinese adolescents and has demonstrated good reliability across three dimensions (36).

ACEs were assessed using the Childhood Trauma Questionnaire-Short Form (CTQ-SF) (37). The CTQ-SF comprises 28 items that evaluate five dimensions: emotional abuse, physical abuse, sexual abuse, emotional neglect, and physical neglect. Each item is rated on a 5-point scale from 1 to 5, with total scores ranging from 28 to 140. A higher total score indicates a more severe level of childhood trauma. In this study, moderate to severe childhood trauma was defined as meeting at least one of the following criteria: emotional abuse ≥ 13, physical abuse ≥ 10, sexual abuse ≥ 8, emotional neglect ≥ 15, and physical neglect ≥ 10 (37). The scale has been shown to possess good reliability and validity in China (9).

Adolescent learning burnout was assessed using the Adolescent Learning Burnout Scale, which consists of 16 items encompassing three dimensions: physical and mental exhaustion, academic alienation, and low achievement (38). Each item is rated on a 5-point scale from 1 to 5, with total scores ranging from 16 to 80. A higher total score indicates a greater severity of learning burnout. This scale has been widely employed in related research and has demonstrated good reliability and validity (39).

Depression was evaluated using the Patient Health Questionnaire Depression Scale-9 item (PHQ-9), which assesses the severity of depressive symptoms experienced over the past two weeks (40). The PHQ-9 consists of 9 items, each rated on a 4-point scale from 0 to 3, with total scores ranging from 0 to 27. A higher total score indicates a more severe level of depression.

### Data analysis

Data analysis was conducted using SPSS 26.0. Descriptive statistics, including frequency, mean, and standard deviation, were utilized to characterize the sample. The Kolmogorov-Smirnov test was employed to determine whether the measurement data followed a normal distribution. Harman’s single-factor test was conducted to assess common method bias, and Pearson correlation analysis was used to examine the relationships among the scores of various scales. Structural equation modeling was constructed using Amos 24.0 to investigate the relationships among parenting styles, ACEs, learning burnout, and depression. Standardized regression coefficients were calculated to derive path coefficients, and fit indices were employed to evaluate the goodness of fit between the data and the hypothesized model, including the standard chi-square (χ^2^/df), root mean square error of approximation (RMSEA ≤ 0.06), adjusted goodness of fit index (AGFI ≥ 0.90), goodness of fit index (GFI ≥ 0.90), comparative fit index (CFI ≥ 0.90), normed fit index (NFI ≥ 0.90), and incremental fit index (IFI ≥ 0.90) (41–43). The bootstrapping method was utilized to test the statistical significance of the direct, indirect, and total effects of the hypothesized model.

## Results

### Characteristics of the study sample

A total of 3,180 participants were included in this study, with an average age of 14.28 ± 1.63 years. The demographic characteristics of the participants and the results of key variables are presented in **Table 1**.

**Table 1:** Demographic characteristics of the study sample (N = 3180).

Characteristic	Number	Percent (%)
Age (years)	14.28 ± 1.63	
11-15	2429	76.4
16-23	751	23.6
Sex		
Male	1456	45.8
Female	1724	54.2
Grade		
7th	700	22.0
8th	708	22.3
9th	749	23.6
10th	402	12.6
11th	359	11.3
12th	262	8.2
Key variables (mean ± SD)		
rejection and denial (s-EMBU)	24.18 ± 7.16	
emotional warmth and understanding (s-EMBU)	31.25 ± 8.02	
overprotection and interference (s-EMBU)	38.41 ± 8.19	
CTQ-SF	93.83 ± 14.67	
Learning Burnout	51.67 ± 7.90	
PHQ-9	12.42 ± 3.28	

### Correlation coefficient of key variables using Pearson correlation among participants

**Table 2** indicates a significant relationship between adolescent depression and parental upbringing styles. Specifically, parenting styles characterized by rejection and denial (*r* = 0.131, *p* < 0.01) as well as overprotection and interference (*r* = 0.518, *p* < 0.01) are positively correlated with depression. Additionally, ACEs (*r* = 0.193, *p* < 0.01) and learning burnout (*r* = 0.246, *p* < 0.01) also exhibit a positive correlation with depressive symptoms. Conversely, parenting styles that emphasize emotional warmth and understanding are negatively correlated with depression (*r* = -0.252, *p* < 0.01). Furthermore, parental emotional warmth and understanding are negatively associated with ACEs (*r* = -0.389, *p* < 0.01) and learning burnout (*r* = -0.162, *p* < 0.01). In contrast, rejection and denial, as well as overprotection and interference, are positively correlated with ACEs (*r* = 0.576, *p* < 0.01; *r* = 0.263, *p* < 0.01) and learning burnout (*r* = 0.041, *p* < 0.01; *r* = 0.056, *p* < 0.01).

**Table 2:** Correlations of all the key variables.

Variables	1	2	3	4	5	6
1 RD	1					
2 EWU	-0.252^**^	1				
3 OI	0.518^**^	0.005	1			
4 ACEs	0.576^**^	-0.389^**^	0.263^**^	1		
5 LB	0.041^*^	-0.162^**^	0.056^**^	0.082^**^	1	
6 DP	0.131^**^	-0.100^**^	0.077^**^	0.193^**^	0.246^**^	1

^**^ *p* < 0.01; RD, rejection and denial; EWU, emotional warmth and understanding; OI, overprotection and interference; ACEs, adverse childhood experiences; LB, learning burnout; DP, depression.

### Structural equation model

**Figure 3:**
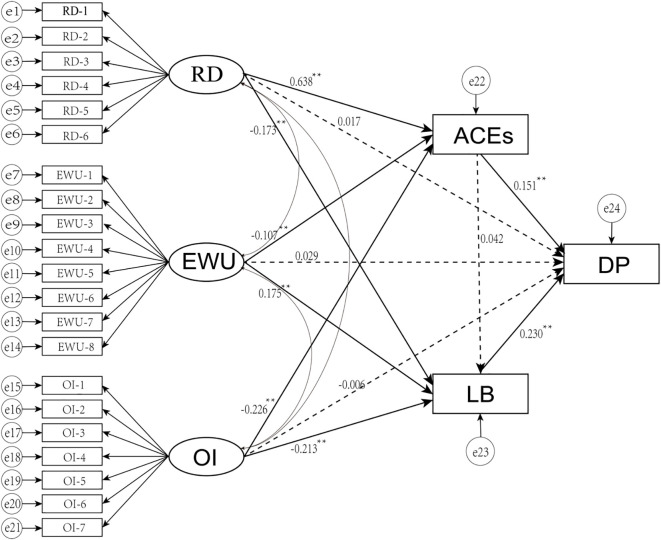
The SEM on mediating effect of adverse childhood experiences and learning burnout between parting styles and depression. RD, rejection and denial; EWU, emotional warmth and understanding; OI, overprotection and interference; ACEs, adverse childhood experiences; LB, learning burnout; DP, depression. All the path coefficients are standardized.

SEM was established in this study to examine the relationships among various parenting styles, ACEs, learning burnout, and depression (**Table 3**, **Figure 3**). The results indicate that the model fit indices met the acceptable standards: χ²/df = 8.689, GFI = 0.955, AGFI = 0.938, CFI = 0.923, NFI = 0.914, IFI = 0.923, and RMSEA = 0.049.

**Table 3:** Model fit indices of mediating effect of adverse childhood experiences and learning burnout between parenting styles and depression.

χ2/df	RMSEA	GFI	AGFI	CFI	NFI	IFI
8.689	0.049	0.955	0.938	0.923	0.914	0.923

### Path coefficient

The standardized path coefficients (*β*) from the SEM model are summarized in **Table 4**. Statistically significant were found from various parenting styles to ACEs, and from ACEs to depression (Rejection and Denial [RD]: *β* = 0.638, Emotional Warmth and Understanding [EWU]: *β* = -0.107, Overprotection and Intrusion [OI]: *β* = -0.226, *p* < 0.05; ACEs to depression: *β* = 0.151, *p* < 0.05). Similarly, the paths from different parenting styles to learning burnout and from learning burnout to depression were also significant (RD: *β* = -0.173, EWU: *β* = 0.175, OI: *β* = -0.213, *p* < 0.05; learning burnout to depression: *β* = 0.230, *p* < 0.05). However the direct effects of different parenting styles on depression and the path from ACEs to learning burnout were not statistically significant (RD: *β* = 0.017, EWU: *β* = 0.029, OI: *β* = -0.006, *p* > 0.05; ACEs to learning burnout: *β* = 0.042, *p* > 0.05).

**Table 4:** Path coefficient of SEM.

Assuming path	Estimate (std.)	S.E.	C.R.	*P*	Hypothesis
ACEs<—RD	0.638	0.401	17.475	0.000	Supported
ACEs<—EWU	-0.107	0.330	-3.273	0.001	Supported
ACEs<—OI	-0.226	0.191	-11.249	0.000	Supported
LB<—RD	-0.173	0.355	-3.723	0.000	Supported
LB<—EWU	0.175	0.280	4.389	0.000	Supported
LB<—OI	-0.213	0.163	-8.655	0.000	Supported
DP<—RD	0.017	0.142	0.390	0.697	Not supported
DP<—EWU	0.029	0.112	0.760	0.447	Not supported
DP<—OI	-0.006	0.065	-0.252	0.801	Not supported
DP<—ACEs	0.151	0.007	6.020	0.000	Supported
DP<—LB	0.230	0.007	13.173	0.000	Supported
LB<—ACEs	0.042	0.018	1.597	0.110	Not supported

RD, rejection and denial; EWU, emotional warmth and understanding; OI, overprotection and interference; ACEs, adverse childhood experiences; LB, learning burnout; DP, depression.

### Mediation analysis

Further assessment of the mediating effects of ACEs and learning burnout on the relationship between parenting styles and depression was conducted through bootstrap resampling (**Table 5**). The path coefficients from parenting styles characterized by rejection and denial, emotional warmth and understanding, and overprotection and interference to depression via ACEs were 0.305, -0.047, and -0.094, respectively (*p* < 0.05). These results indicate that ACEs significantly mediates the relationship between various parenting styles and depression, thereby supporting the validity of Path 1 in Hypothesis 1. Additionally, the path coefficients from the aforementioned parenting styles to depression through learning burnout were -0.127, 0.118, and -0.135, respectively (*p* < 0.05). This suggests that learning burnout also significantly mediates the relationship between different parenting styles and depression, confirming the validity of Path 2 in Hypothesis 1. However, the mediating effects of ACEs and learning burnout on depression through parenting styles characterized by rejection and denial, emotional warmth and understanding, and overprotection and interference were found to be non-significant, thereby refuting Path 3 in Hypothesis 2.

**Table 5:** Intermediary effect test.

Assuming path		Estimate	Lower	Upper	*P*	Hypothesis
Ind1	RD–>ACEs–>DP	0.305	0.203	0.420	0.000	Supported
Ind2	EWU–>ACEs–>DP	-0.047	-0.088	-0.019	0.001	Supported
Ind3	OI–>ACEs–>DP	-0.094	-0.135	-0.060	0.000	Supported
Ind4	RD–>LB–>DP	-0.127	-0.206	-0.058	0.000	Supported
Ind5	EWU–>LB–>DP	0.118	0.064	0.183	0.000	Supported
Ind6	OI–>LB–>DP	-0.135	-0.178	-0.099	0.000	Supported
Ind7	RD–>ACEs–>LB–>DP	0.019	-0.004	0.047	0.105	Not supported
Ind8	EWU–>ACEs–>LB–>DP	-0.003	-0.009	0.000	0.077	Not supported
Ind9	OI–>ACEs–>LB–>DP	-0.006	-0.014	0.001	0.095	Not supported

### Gender and parental differences in the model

To investigate whether there are differences in the pathways through which paternal and maternal parenting styles influence depression, we incorporated both parenting styles into our model. The results indicated that there were no significant differences in the effects of paternal and maternal parenting styles on depression. ACEs and learning burnout served as mediators in the relationship between parenting styles and depression. The path coefficients from ACEs to learning burnout were statistically significant (*β* = 0.059, *β* = 0.061; *p* < 0.05, **Tables 6**, **7**), demonstrating a chain mediation effect between ACEs, learning burnout, and depression for both paternal and maternal parenting styles. This finding disproves Hypothesis 3.

**Table 6:** Path coefficient of parenting style.

Assuming path	Estimate (std.)	S.E.	C.R.	*P*	Hypothesis
ACEs<—RD	0.554	0.742	13.686	0.000	Supported
ACEs<—EWU	-0.095	0.631	-2.582	0.010	Supported
ACEs<—OI	-0.159	0.393	-7.101	0.000	Supported
LB<—RD	-0.183	0.617	-3.77	0.000	Supported
LB<—EWU	0.160	0.505	3.795	0.000	Supported
LB<—OI	-0.194	0.315	-7.497	0.000	Supported
DP<—RD	0.004	0.246	0.094	0.925	Not supported
DP<—EWU	0.028	0.201	0.681	0.496	Not supported
DP<—OI	-0.008	0.126	-0.34	0.734	Not supported
DP<—ACEs	0.160	0.006	7.339	0.000	Supported
DP<—LB	0.230	0.007	13.213	0.000	Supported
LB<—ACEs	0.059	0.016	2.594	0.009	Supported

**Table 7:** Path coefficient of maternal parenting style.

Assuming path	Estimate (std.)	S.E.	C.R.	*P*	Hypothesis
ACEs<—RD	0.513	0.783	12.807	0.000	Supported
ACEs<—EWU	-0.081	0.63	-2.278	0.023	Supported
ACEs<—OI	-0.179	0.406	-7.657	0.000	Supported
LB<—RD	-0.199	0.632	-4.266	0.000	Supported
LB<—EWU	0.179	0.500	4.387	0.000	Supported
LB<—OI	-0.203	0.323	-7.553	0.000	Supported
DP<—RD	0.011	0.252	0.243	0.808	Not supported
DP<—EWU	0.026	0.200	0.661	0.509	Not supported
DP<—OI	0.006	0.129	0.218	0.827	Not supported
DP<—ACEs	0.163	0.006	7.637	0.000	Supported
DP<—LB	0.232	0.007	13.285	0.000	Supported
LB<—ACEs	0.061	0.015	2.753	0.006	Supported

To investigate whether there are differences in the pathways through which paternal and maternal parenting styles influence depression, we incorporated both parenting styles into our model. The results indicated that there were no significant differences in the effects of paternal and maternal parenting styles on depression. ACEs and learning burnout served as mediators in the relationship between parenting styles and depression. The path coefficients from ACEs to learning burnout were statistically significant (*β* = 0.059, *β* = 0.061; *p* < 0.05, **Tables 6**, **7**), demonstrating a chain mediation effect between ACEs, learning burnout, and depression for both paternal and maternal parenting styles. This finding disproves Hypothesis 3.

**Figure 4:**
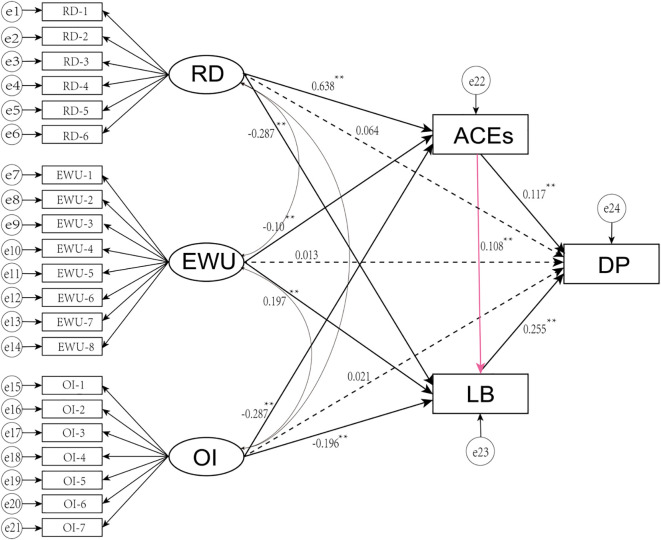
SEM for female adolescents.

**Figure 5:**
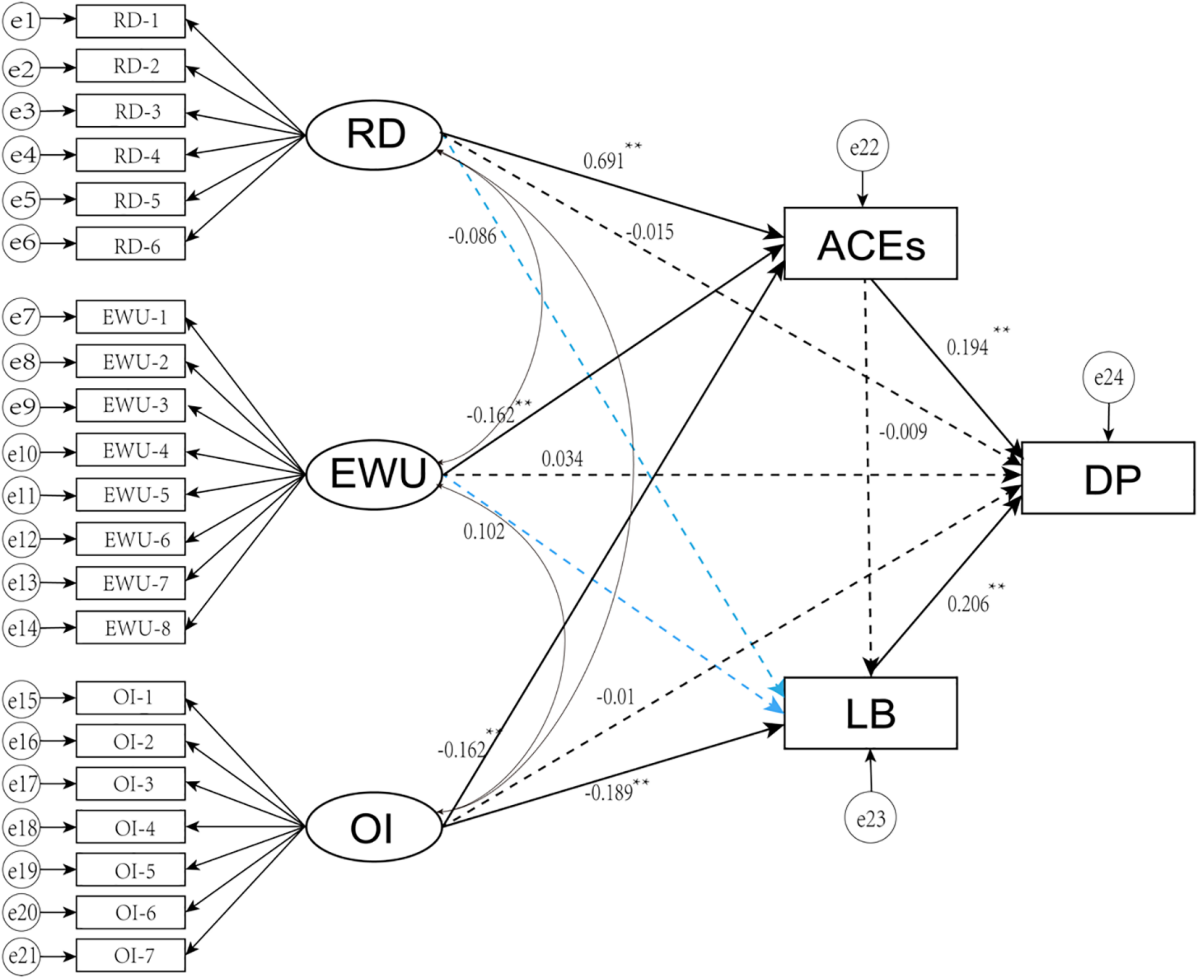
SEM for male adolescents.

To further explore whether there are gender differences in the pathways through which parenting styles affect adolescent depression, we separated participants by gender and incorporated them into the model. Our findings revealed that the mediating effect of ACEs between parenting styles and depression did not differ between males and females. However, for female adolescents, parenting styles characterized by rejection and denial, as well as excessive protection and interference, positively predicted the occurrence of depression through the mediating effect of learning burnout (*β* = -0.183, *β* = -0.148, *p* < 0.05). Conversely, parenting styles that emphasize emotional warmth and understanding negatively predicted depression (*β* = 0.140; *p* < 0.05, **Table 8**, **Figure 4**). In male adolescents, only excessive protection was found to influence depression through the mediating role of learning burnout (*β* = -0.106; *p* < 0.05, **Table 9**, **Figure 5**). Moreover, in female adolescents, parental parenting styles could also influence depression through a chain mediation effect from ACEs to learning burnout, a pathway that was not observed in male adolescents. Therefore, there are indeed differences in the pathways leading to depression resulting from parental parenting styles between genders, thereby supporting Hypothesis 4.

**Table 8:** Path coefficients influencing depression in female adolescents.

Assuming path		Estimate	Lower	Upper	*P*	Hypothesis
Ind1	RD–>ACEs–>DP	0.244	0.08	0.406	0.003	Supported
Ind2	EWU–>ACEs–>DP	-0.032	-0.073	-0.009	0.003	Supported
Ind3	OI–>ACEs–>DP	-0.100	-0.17	-0.034	0.003	Supported
Ind4	RD–>LB–>DP	-0.183	-0.298	-0.087	0.001	Supported
Ind5	EWU–>LB–>DP	0.140	0.077	0.213	0.000	Supported
Ind6	OI–>LB–>DP	-0.148	-0.209	-0.097	0.000	Supported
Ind7	RD–>ACEs–>LB–>DP	0.057	0.016	0.106	0.005	Supported
Ind8	EWU–>ACEs–>LB–>DP	-0.008	-0.019	-0.002	0.004	Supported
Ind9	OI–>ACEs–>LB–>DP	-0.023	-0.042	-0.007	0.005	Supported

**Table 9:** Path coefficients influencing depression in male adolescents.

Assuming path		Estimate	Lower	Upper	*P*	Hypothesis
Ind1	RD–>ACEs–>DP	0.429	0.255	0.656	0.000	Supported
Ind2	EWU–>ACEs–>DP	-0.095	-0.227	-0.022	0.009	Supported
Ind3	OI–>ACEs–>DP	-0.086	-0.149	-0.036	0.001	Supported
Ind4	RD–>LB–>DP	-0.057	-0.195	0.054	0.325	Not supported
Ind5	EWU–>LB–>DP	0.063	-0.031	0.179	0.183	Not supported
Ind6	OI–>LB–>DP	-0.106	-0.175	-0.054	0.001	Supported
Ind7	RD–>ACEs–>LB–>DP	-0.004	-0.043	0.035	0.810	Not supported
Ind8	EWU–>ACEs–>LB–>DP	0.001	-0.008	0.012	0.719	Not supported
Ind9	OI–>ACEs–>LB–>DP	0.001	-0.006	0.009	0.782	Not supported

## Discussion

This study aims to examine the relationship between various parenting styles and depression, as well as the roles of ACEs and learning burnout in this context. The findings indicate that both ACEs and learning burnout serve as complete mediators in the relationship between different parenting styles and depression. There are no significant differences in the pathways leading to depression between paternal and maternal parenting styles. However, it was further discovered that for female adolescents, negative parenting styles can lead to the onset of depression through both the mediating and chain mediating effects of ACEs and learning burnout. In contrast, for male adolescents, ACEs continues to play a mediating role in the relationship between negative parenting styles and depression, while only overprotective parenting styles influence the occurrence of depression through the mediating effect of learning burnout.

The results of this study indicate that negative parenting styles can influence adolescent depression through ACEs or learning burnout, which is consistent with previous research findings (15, 33, 44, 45). Negative parenting practices, such as rejection, denial, and neglect—exemplified by a lack of protection and care for the child, indifference to emotional needs, and humiliation or verbal abuse—can lead children to experience feelings of neglect or even abuse, thereby creating ACEs. Consequently, these children may struggle to establish secure attachment relationships and cope with life stressors and setbacks, resulting in an increased risk of negative experiences and, ultimately, depression (46, 47). Within the context of China’s highly competitive educational system, parenting styles often prioritize academic achievement (48, 49). Not all parents adopt positive parenting strategies to enhance adolescents’ motivation for learning; instead, some may establish authority through denial, rejection, and excessive interference to stimulate academic drive (44). However, the prolonged absence of emotional support and understanding, coupled with the deprivation of autonomy and choice, makes adolescents more susceptible to negative emotional experiences and task avoidance strategies when faced with intense academic pressure, leading to learning burnout. Prolonged exposure to excessive burnout can disrupt the functioning of the HPA axis, resulting in increased cortisol secretion, which in turn can impair the prefrontal cortex and elevate the risk of depression (1). Furthermore, the findings of this study reveal a chain mediation effect of ACEs and learning burnout in the relationship between parenting styles and depression. Negative parenting practices can lead to psychological issues such as insecure emotional attachment, a lack of self-worth, negative thought patterns, and difficulties in emotional regulation, making adolescents more vulnerable to the impact of setbacks and challenges, thereby contributing to the occurrence of ACEs (50). Previous research has confirmed that negative parenting styles and ACEs have detrimental effects on children’s neurodevelopment (51, 52). Long-term exposure to negative parenting can adversely affect adolescent neurodevelopment, such as prolonged increases in cortisol levels and reductions in prefrontal cortex gray matter volume, which in turn restricts learning and executive functions (53), leading to learning burnout and an increased risk of depression.

Although this study focuses on the influence of negative parenting styles on adolescent depression through ACEs and learning burnout, recent empirical research in the Asian context suggests that the negative effects extend beyond depressive symptoms. A study involving Indonesian high school students (54) found that authoritarian parenting significantly elevated adolescents’ callous-unemotional (CU) traits, which have been confirmed by numerous longitudinal studies as stable precursors to antisocial behavior and violent crime. This indicates that the same negative parenting model can simultaneously instigate two risk trajectories—depression and antisocial behavior—across different individuals or subgroups: on one hand, it triggers “internalizing” issues (such as hopelessness, sleep disorders, and learning burnout) through cumulative ACEs, while on the other hand, it generates “externalizing” problems (such as bullying, misconduct, and callousness) due to impaired emotional recognition and prosocial motivation. Given that depression has been established as the strongest predictor of suicidal ideation globally (55), clarifying its developmental pathways is crucial for suicide prevention. Therefore, a comprehensive approach that includes multidimensional screening (for depression, callousness, and impulsivity) and multilayered interventions (such as parenting training, school emotional curricula, and community peer support) is essential to effectively mitigate the long-term impacts of negative parenting on adolescents’ mental health and behavioral adaptation, ultimately reducing the incidence of suicide.

In this study, gender differences in parenting styles were found to influence the pathways leading to the onset of depression. Specifically, female adolescents exhibit a greater number of pathways impacting the occurrence of depression compared to their male counterparts. Numerous prior studies have established that females are more susceptible to depression than males (56, 57). Hyde et al. (58) based on the vulnerability-stress model, proposed the ABC (Affective, Biological, Cognitive) model, which posits that, in comparison to males, females experience cognitive vulnerabilities (such as negative attribution styles, greater rumination on negative events, and negative beliefs about their bodies), emotional vulnerabilities (including low attention, low rhythmicity, and low adaptability), and biological vulnerabilities (characterized by sex differentiation in the brain, sex-specific genetic loci, and hormonal levels). These three types of vulnerabilities interact with adverse life events, thereby increasing the likelihood of depressive symptoms in females. Research has indicated that girls report a higher incidence of negative life events than boys, including experiences of childhood sexual abuse, negative interpersonal relationships, and peer sexual harassment, which further suggests that females are more likely to develop depression as a result of the interplay between vulnerabilities and adverse life events (56). The findings of this study indicate that, among female adolescents, negative parenting styles can lead to the onset of depression through both direct and chain mediation effects involving ACEs and learning burnout. This further corroborates the notion that females are more susceptible to the influences of environmental factors and life events in relation to the development of depression compared to males. Consequently, interventions for female adolescents must be multifaceted, necessitating not only adjustments in parenting styles but also the provision of trauma-informed support and strategies to address learning burnout within educational settings.

Interestingly, this study reveals that male adolescents exhibit heightened sensitivity to overprotective parenting styles. The adolescent period is characterized by identity exploration and the development of independence; however, parental overprotection can hinder the establishment of a distinct identity and autonomy. This may adversely affect their self-esteem and self-identity, leading male adolescents to exhibit tendencies toward rebellion and a pursuit of autonomy as a means to counteract the constraints imposed by overprotection (59). From the perspective of social role expectations, society anticipates males to be more independent and resilient. Boys raised in overprotective environments often lack autonomy and a sense of responsibility, as well as the experience necessary to cope with setbacks and challenges. Consequently, male adolescents may feel unable to meet societal expectations, which can exacerbate anxiety and stress, ultimately negatively impacting their mental health (60). The findings of this study indicate that, among male adolescents, overprotective parenting styles can influence the onset of depression through the mediating role of learning burnout. In other words, when parenting male adolescents, it is crucial for parents to focus on providing support and encouraging autonomy, as this approach may help prevent or mitigate the occurrence of depression.

This study, through structural equation modeling analysis, reveals significant gender differences in the chain mediation pathway whereby parenting styles influence adolescent depression through ACEs and learning burnout. This pathway is established within the female cohort, while it does not hold for the male cohort. Such discrepancies may arise from a confluence of factors. Firstly, from a socio-psychological development perspective, female adolescents typically exhibit heightened sensitivity to familial emotional environments and interpersonal interactions (61). Consequently, parenting styles, such as rejection and overprotection, may exert a more direct influence on children’s emotional cognition and stress perception, thereby precipitating ACEs at an earlier developmental stage. This, in turn, manifests as learning burnout and depressive symptoms. In contrast, male adolescents may demonstrate a stronger response to external environments (such as peer relationships and academic performance), with the influence of parenting styles on their psychological pathways potentially mitigated or obscured by other socialization factors. Additionally, the impact of statistical power cannot be disregarded; if the sample size for males is relatively small, the chain mediation effect may remain undetected due to insufficient statistical power. Future research should further elucidate the nature of this pathway difference through multi-group comparative analyses and gender-stratified examinations with larger sample sizes.

This study focuses on high-risk adolescents with moderate to severe childhood trauma experiences and mild to moderate depressive symptoms. This demographic is particularly significant due to the severe challenges they face in psychological development, as their depressive symptoms often manifest earlier, persist for longer durations, and are more likely to evolve into severe psychological disorders. Understanding the pathways that contribute to the depressive risk among high-risk adolescents can facilitate the design of targeted intervention strategies for families and schools, enhancing early identification and intervention efforts.

## Limitations

This study has several limitations. Firstly, the participants were drawn from a specific district in Chengdu, which may limit the generalizability of the findings. External factors such as socioeconomic status and family structure could also influence the results. Future research should consider incorporating these variables and recruit samples from a broader range of regions, including a general adolescent population, to enhance the applicability of the findings. Secondly, the assessment of psychological scales relied entirely on self-reports, which are susceptible to subjective biases that may compromise the objectivity of the results. To ensure the objectivity and authenticity of future findings, subsequent studies should incorporate additional objective measures, such as physiological indicators and neuroimaging metrics.

## Conclusions

Research based on a sample of high-risk adolescents has revealed that ACEs and learning burnout serve as mediating factors between parenting styles and depressive symptoms. This mediating pathway exhibits gender differences; females demonstrate greater sensitivity to various negative parenting practices, resulting in a more complex relationship, while males show a stronger response to overprotective and intrusive parenting styles. These findings suggest that it is essential to address risk factors specific to each gender in order to mitigate the risk of depression among adolescents. However, the reliance on a single urban sample limits the external validity of these results, necessitating further research to corroborate these findings.

## Data Availability

The original contributions presented in the study are included in the article/**Supplementary Material**. Further inquiries can be directed to the corresponding author.
